# Systematic review on the effects of the discontinuation of the anticoagulant therapy and the postoperative bleeding, in patients under new oral anticoagulants after dental extraction

**DOI:** 10.4317/jced.60122

**Published:** 2023-04-01

**Authors:** Mónica López-Galindo, María Grau-Benítez

**Affiliations:** 1 Associate Professor. PhD. Dentistry Department. Faculty of Health Sciences. Universidad Europea de Valencia, Spain

## Abstract

**Background:**

The present systematic review compares the effects of the discontinuation of the anticoagulant therapy and the postoperative bleeding, in patients under new oral anticoagulants after dental extraction. The purpose of this study is to determine the postoperative complications of the DOACs after a simple dental extraction in comparison to the VKAs and with patients not under anticoagulants. This study aims to determine the postoperative complications of the DOACs in the case of an alteration of the anticoagulant regimen before a dental extraction.

**Material and Methods:**

The electronic search was conducted on two databases (MedLine complete and Scopus). The research included patients under DOACs undergoing simple dental extraction. The inclusion criteria included randomized controlled trials, cohort studies, case series, retrospective cohort, prospective cohort and studies on human individuals.

**Results:**

7 studies were selected, complying to all the inclusion criteria. 931 patients were treated. The bleeding rate was ranging from none, minor, moderate. All the studies included a postoperative follow up from the day of the surgery. The bleeding is immediate and minor after a dental extraction for patients under DOACs, VKAs and with no-OAT.

**Conclusions:**

The most frequent postoperative complication for patients under DOACs and patients under VKAs after a simple dental extraction is minor bleeding: immediate or delayed. DOACs seem to be a safe drug and do not require the discontinuation/alteration of the therapy for a simple dental extraction. Further studies are required to determine if surgical procedures in dentistry require an alteration of the DOAC regimen.

** Key words:**New oral anticoagulants, NAOCs, DOACs, VKAs, Exodontia, Simple extraction, Dabigatran, Rivaroxaban, Apixaban, Edoxaban, Postoperative bleeding.

## Introduction

The oral anticoagulant therapy (OAT) is an increasingly common treatment in the population. In the dental field, it is essential to monitor the patients taking anticoagulants, in order to achieve the adequate protocol as treatment. To prevent the clot from spreading inappropriately, there are natural anticoagulants, called inhibitors, which are also produced by the liver: antithrombin, protein C, protein S, etc. However, in some diseases, the coagulation is altered, leading to hematopoietic diseases, such as thromboembolism. To treat and prevent these abnormalities and inhibit the coagulation system more selectively, anticoagulants will be prescribed (1). They can be administered orally or intravenously.

There are two types of oral anticoagulants: VKAs (Vitamin K Antagonists) and NOACs (Novel Oral Anticoagulants): Oral antivitamin K anticoagulants are used in atrial fibrillation (valvular or non-valvular) and direct-acting oral anticoagulants (DOA) are used in non-valvular atrial fibrillation (1). The patients under a NOACs have to be under daily medication. (2) The first new oral anticoagulant was developed in 2010: Dabigatran. Followed by Rivaroxaban (2011), Apixaban (2012), Edoxaban (2014). (3) These drugs are prescribed more and more over the years, due to the aging of the population. Nevertheless, until today, many health professionals are not aware of the management of the patients under new oral anticoagulants since it is a new drug on the market that differs a lot from the conventional ones (i.e. VKAs or Heparin) in its use and its dosage. It is a new drug for dentists and more studies are required in order to study the complications and the management of patients under new oral anticoagulants in order to be prepared before the invasive procedures. A few guidelines are available on many databases; however, many doubts are still present towards these new drugs. One of the daily treatments at a dentist is the simple dental extraction. It is also an invasive procedure, although presenting minimal risks as long as it is performed in an aseptic area, respecting the hemostatic procedures. However, in case of a patient under anticoagulants, this procedure can present many risks and perioperative complications that the dentist should be aware of. For all the aforementioned, the aim of the present review is to evaluate the possible discontinuation/alteration of the anticoagulant therapy and the postoperative bleeding of patients under new oral anticoagulants after a simple dental extraction was performed in order to analyze the issues derived.

## Material and Methods

- Protocol and focused question: The present systematic review was carried out according to the PRISMA (Preferred Reporting Items for Systematic Reviews and Meta-Analyses) (4). The following focus question was employed according to the population, intervention, comparison, and outcome study design: Among anticoagulant patients undergoing simple dental extraction, do direct oral anticoagulants demonstrate a reduction of the intra- and postoperative complications in comparison to VKAs anticoagulants? 

-Selection criteria: Before starting the study, a series of inclusion and exclusion criteria were established. Patients taking dabigatran, rivaroxaban, apixaban or edoxaban undergoing a simple dental extraction were included. The types of studies included randomized controlled trials; cohort studies; case series; retrospective cohort; prospective cohort; studies on human individuals; publications in English or Spanish; studies about more than 5 patients; published until December 2021. The variable included studies that will provide data related to assessing bleeding risk after a simple dental extraction in patients undertaking NOACs with or without altering the anticoagulation therapy. And studies comparing the bleeding risk and the complications of the NOACs with the VKAs. Studies about patients taking intravenous anticoagulants, patients undergoing 

other dental treatment, and studies published before 2011 were excluded.

-Search strategy: An electronic search was carried out by the authors in two databases: Medline complete and Scopus including articles until December 2021. The following keywords were combined with the Boolean AND and OR operators, as well as the controlled terms (such as the “MeSh” words) in an attempt to obtain the best and broadest search: “((simple dental extraction) OR (Dental extraction) OR (exodontia) OR (dentoalveolar surgery) OR (Tooth extraction) OR (surgery,oral)) AND ((direct oral anticoagulants) OR (oral anticoagulants) OR (new oral anticoagulants) OR (novel oral anticoagulants) OR (non-vitamin K oral anticoagulants) OR (NVKA oral anticoagulants) OR (NOACs) OR (DOACs) OR (anticoagulants) ) AND ( (vitamin K antagonists anticoagulants) OR (conventional anticoagulants) OR (VKAs anticoagulants) OR (anticoagulants)) AND ((intraoperative complications) OR (postoperative complications) OR (complications) OR (bleeding) OR (bleeding time) OR (risk factors))” The search was completed with a review of the references provided in each of the studies in order to identify any additional studies. Finally, a cross-search of potentially interesting articles was carried out for the introduction.

-Screening methods and data abstraction: The selection of the studies was carried out by two reviewers (AH, ML). A selection process was carried out. The titles were reviewed in order to eliminate irrelevant publications, followed by the removal of the duplicated articles. The abstracts were reviewed and filtered according to the type of study, type of invasive treatment, type of extraction, type of anticoagulants, and result variables. The last stage consisted of a complete reading of each article and extracting the data according to a predetermined data extraction form to confirm the eligibility of the studies. In case of any disagreement, it was resolved by mutual consensus of both the reviewers. The following information was extracted from the studies: Authors detail, year of publication, country, study design, age and gender of the patients, type of DOAC, other oral anticoagulants (VKAs), study size, number of teeth extracted, teeth extraction method, hemostatic measures, bleeding rate, interruption of DOAC or not, follow-up time post extraction. These data were independently extracted.

- Risk of bias in individual studies: The quality of the included studies was assessed by two reviewers (AH, ML) in order to assess the methodological quality and risk of bias of all included articles. The methodological quality of the studies was performed according to the CASPE guideline (5). Studies were considered to be at “low risk of bias” if they me*et al*l the criteria. If there was a possible bias in at least one criteria, they were considered to have an “uncertain risk of bias”. And the studies with a “high risk of bias” were those where one or more criteria were not met or with doubts in more than one criteria. The degree of agreement regarding the evaluation of the quality of the studies was obtained with the Cohen kappa test, following the Landis and Koch scale (6).

- Case definitions: A simple dental extraction is the removal of a tooth that is fully erupted. For this treatment, the patient is anesthetized, to numb the area and reduce the pain. The dentist uses elevators and dental forceps as instruments in order to elevate the tooth and grasp the crown of the tooth. No flap opening or suture is required in a simple dental extraction, also called non-surgical dental extraction (7).

## Results

-Study selection: A total of 1034 articles were obtained from the initial search process: Medline Complete (n=332) and Scopus (n=732). Among the 1034 articles, 90 were excluded because they were duplications and 930 were excluded since they were not identified as potentially eligible articles through the screened by titles and abstracts and the inclusion criteria. Another screening was carried out according to the type of study and 7 articles were excluded. As a result, 7 articles m*et al*l the inclusion criteria and were included in the present systematic review. The k-value for agreement between the interexaminer on the study inclusion was 1.0 (titles and abstracts) and 1.0 (study type), indicating “complete” agreement, respectively, based on the criteria from Landis and Koch (6).

- Characteristics of included studies ( Table 1, Table 2): Of the 7 articles included in this review, there was 1 case series (8), 1 prospective observational study (9), 1 prospective case-control study (10), 3 cohort studies (11-14) and 1 prospective comparative study (12). A total of 931 patients were treated, with an average age of 70 years and a majority of them were male. A total of 1795 teeth were extracted: 375 on patients taking DOACs, 305 under VKAs and 1115 on non-OAT patients. There was one study comparing apixaban, dabigatran, edoxaban and rivaroxaban with VKAs and one study comparing VKAs and DOACs in general. Non-OATs patients were compared with patients under DOACs in one study and with patients under DOACs and patients under VKAs in another study. In two studies the patients underwent surgical and simple dental extractions but the overall percentage of surgical extractions was inferior to 15%. In all the included studies elevators and forceps were used for the extraction while performing an atraumatic extraction without cutting the surrounding bone nor cutting the gum, followed by the complete curettage of the inflamed granulation tissue. After this, hemostatic measures were taken. There were two studies (10,14) in which the hemostatic measures were suturing after the compression with gauze. In two studies (8,9) absorbable hemostatic sponges such as oxidized cellulose, gelatin sponge was used in addition to suturing in all the patients, whilst in three studies (11-13) the use of absorbable hemostats was only performed if there as a lack of adequate bleeding control. The sutures were performed after the extractions with non-absorbable material as 4-0 silk, 4.0 polyglactin 910 sutures, Vicryl® 3-0, 3.0 nylon. These sutures were performed for every case except in two studies (11,13) in which the suturing was not performed in every case. The oral anticoagulant therapy with DOACs was discontinued before the extraction in two studies (10,13). The dose of NOAC was skipped on the morning of the procedure in the first study (10) and was restarted according to the normal regimen of the treatment, at least 4 h after the dental extraction following the achievement of an adequate hemostasis. In the other study (13), the perioperative cessation of the NOAC therapy was ranging from 1 to 14 days. In the majority of the studies the bleeding rate was ranging from none, minor, moderate to severe. The bleeding rate was not described in one study (11), however it was mentioned that all the post extraction bleeding could have been stopped with local hemostatic treatment, which represents a minor bleeding rate. The 7 studies included a postoperative follow up from the day of the surgery from 30min after the extraction up to 2 weeks after.

**Table 1:**
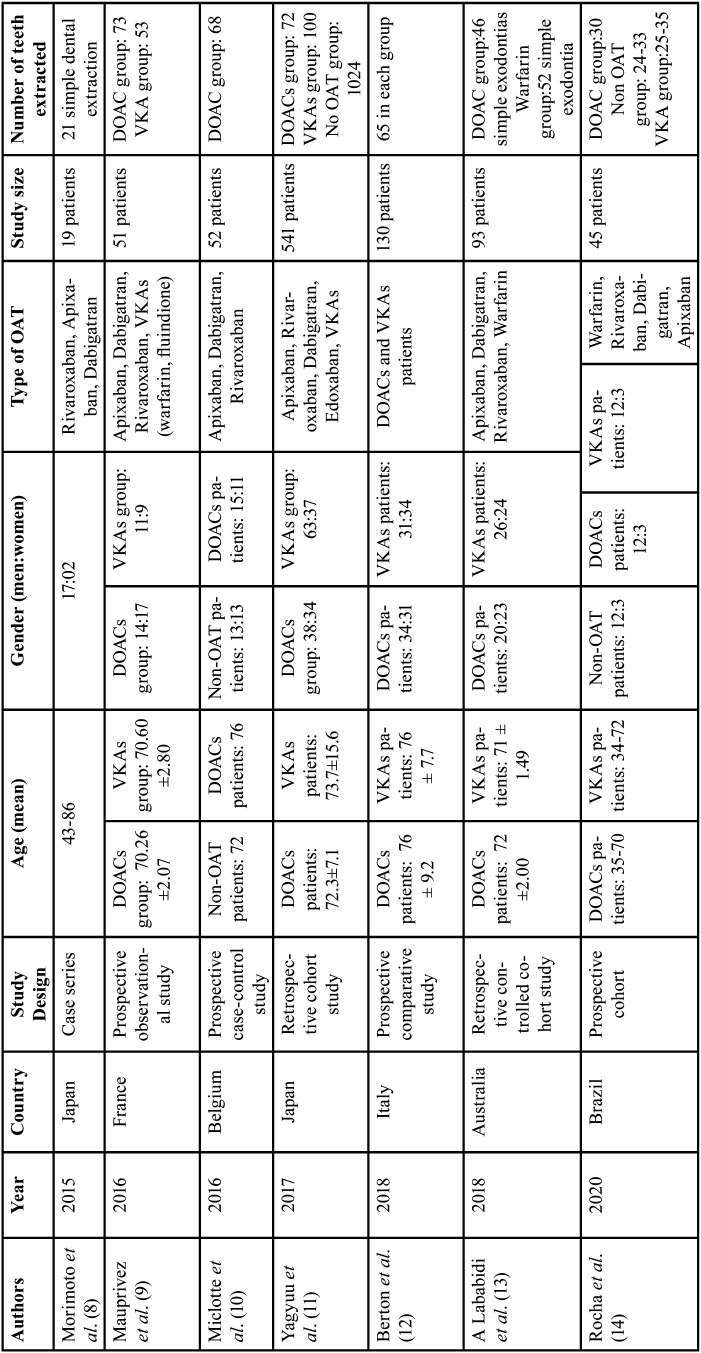
General sample characteristics of the included studies.

**Table 2:**
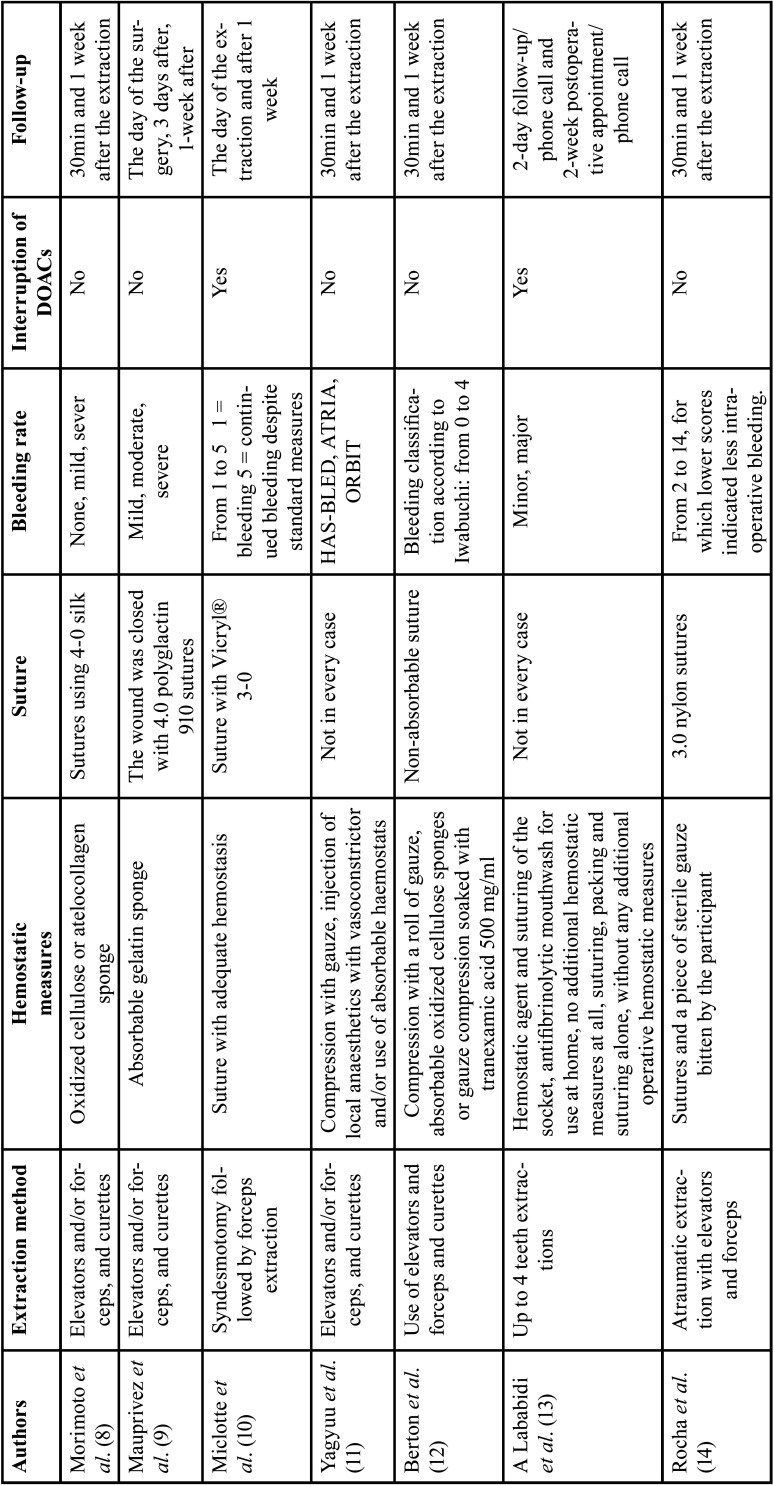
General sample characteristics of the included studies.

- Risk of bias across studies: For the reviewed studies, an uncertain risk of bias was considered in all 7 studies. For the one case series study, it was considered to have a high risk of bias due to the very nature of the type of study. The k value (Cohen kappa test) on the agreement between the reviewers of the methodological quality was 0.80 according to the Landis & Koch scale (6).

- Synthesis of the results: Among the selected articles, five studies (8,10-13) were comparing the complications of the DOACs and VKAs after a dental extraction. The present studies have shown that the most common postoperative complication after a simple dental extraction was immediate postoperative bleeding. Being mostly minor in both groups, there was no significant difference in the immediate postoperative bleeding rate for the DOAC and VKA group after tooth extraction as detailed in Table 3.

**Table 3:**
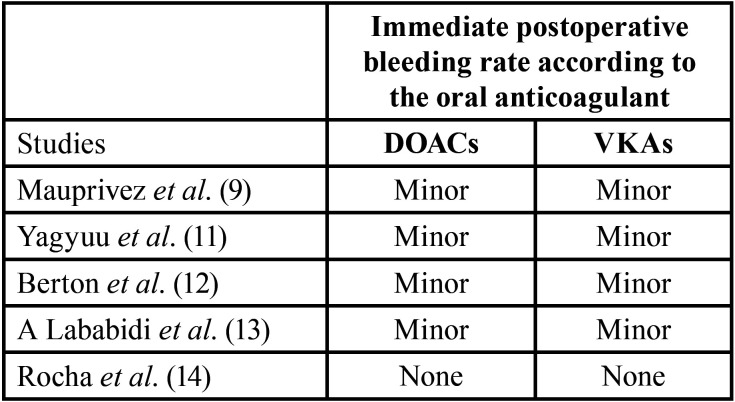
Comparison of the immediate postoperative bleeding rate after dental extraction according to the oral anticoagulant.

Furthermore, another complication encountered was delayed bleeding (Table 4) in the majority of the studies. Overall, delayed bleeding was more encountered in patients under VKAs. In order to control the bleeding, additional hemostatic measures were required in three of the present studies. In the study of Rocha *et al*. (14) only the procedures within the VKA group needed extra hemostatic measures after the dental extraction. In the case of the Berton al study (12) both groups required extra hemostatic measures of post extraction.

**Table 4:**
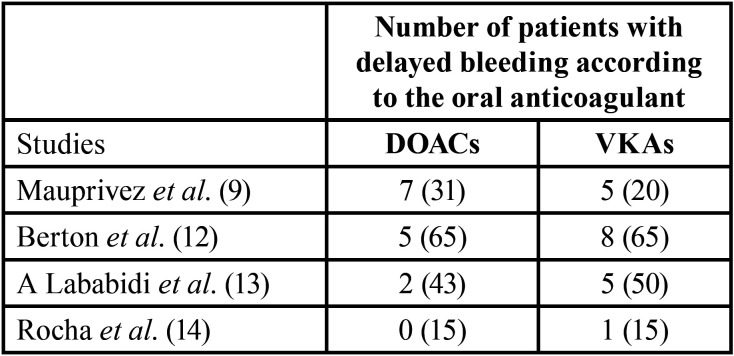
Comparison of the number of patients with delayed postoperative bleeding after simple dental extraction in patients under VKAs and DOACs.

Among the selected articles, three studies (8,10,14) are studying the complications of the patients under DOAC therapy. One article presents the possible complications after tooth extraction for patients under DOAC and the two other studies are comparing the postoperative complications of patients under DOAC therapy and patients not under oral anticoagulant therapy following a simple dental extraction procedure.

In the study of Morimoto *et al*. (8) the postoperative complication was a postoperative minor bleeding rate. However, it is not mentioned if the bleeding rate was considered immediate or delayed. In the studies of Morimoto *et al*. (8) and Miclotte *et al*. (10), all the patients experienced bleeding after the extraction in contrast to the study of Rocha *et al*. (14) where no bleeding happened post-extraction. Furthermore, delayed bleeding was only noticed in the study of Miclotte *et al*. (10). Moreover, postoperative additional hemostatic measures were only carried out in one study (14), where it was required for one procedure in the non-anticoagulated group whilst no additional hemostatic measure was needed in individuals of the DOAC group following the extraction.

In the selected articles, two studies (10,13) included patients with cessation of the morning dose of DOAC the day of the dental extraction.

In the study of A Lababidi *et al*. (13), no bleeding events were observed for these patients, however most of the procedures included additional hemostatic measures following the extraction. Furthermore, in the study of Miclotte *et al*. (10) high bleeding scores were encountered during the procedure for the majority of the patients participating in the study; and the postoperative bleeding -although predominantly minor- was observed immediately after the exodontia but also up to 6 days after the extraction in some cases.

## Discussion

The present systematic review provides information based on the postoperative complications of novel oral anticoagulants after a simple dental extraction. As well as the complications of discontinuation of the anticoagulant therapy for this type of anticoagulant after a simple dental extraction.

Among the postoperative complications of the new oral anticoagulants and the vitamin K antagonists after a simple dental extraction, bleeding is the most frequent complication encountered in the studies (8,10-13). We can encounter two types of bleeding: Immediate and non-immediate/delayed post-operative bleeding. Being encountered in all the studies, the most frequent type of bleeding is the immediate postoperative bleeding. In this review, the bleeding is mild in the two groups of oral anticoagulants. Therefore, both medications present similar immediate complications. In the case of the delayed bleeding post extraction it is not present in all the studies of this review. It is only present in a minority of patients in the studies of Mauprivez *et al*. (9), Berton *et al*. (12), Lababidi *et al*. (13), and Rocha *et al*. (14). There is not a big difference in the number of patients with delayed bleeding after the simple exodontia between the two groups of oral anticoagulants. The study of Wenbing Hua *et al*. (16) has reported that the outcomes for bleeding was less excessive for patients taking DOACs in comparison with the group of VKAs patients and the bleeding risk was also lower. One of the limitations of the present review is that the hemostatic measures are different in the studies, which can influence the bleeding rate. Thereby, the study of Berton *et al*. (12) employed additional hemostatic measures, whereas the study of Lababidi *et al*. (13) either extra hemostatic measures were employed or no additional hemostatic measures at all. However, given the novelty of the DOACs, there are not enough published studies about the complications of anticoagulants related to the dental extractions. And, among the limitations of the present study, the number of eligible patients in the studies is limited since surgical extractions were excluded.

Also, the review was not randomized, which can lead to residual confounding. After a simple dental extraction, all the patients experience bleeding as a complication, whether under DOACs or not under any oral anticoagulant (including antiplatelets). In the present review, the studies of Morimoto *et al*. (8) and Miclotte *et al*. (10) have reported similar complications: minor bleeding immediately after the dental extraction for the patients under DOACS and the patients not under anticoagulants. In contrast to the study of Rocha *et al*. (14) where no postoperative bleeding was experienced except for 1 patient. Another limitation of our results is the complications according to the comorbidities. In the study of Cocero *et al*. (18), the bleeding was compared in patients under DOACs and not under anticoagulants, with and without comorbidities in both groups, in order to identify the risks of bleeding after the extraction in the case of patients under DOACs. In the present review, there is a lack of studies since most of the published articles comparing the patients under DOACs with patients not under anticoagulants include patients under combined anticoagulant therapy (DOAC and VKA or DOAC and antiplatelets) as in the study of Müller *et al*. (19), leading to a lack of evidence on this topic and biased results. Moreover, further studies should include comparison of the complications of every DOAC (Apixaban, Dabigatran, Edoxaban, Rivaroxaban) especially with the low evidence about Edoxaban in the present review since it is only encountered in one study (11). It should also include comparison of the complications according to the indication of the DOACs therapy. The results of the present review also showed that the alteration of the DOACs therapy for the dental extraction, does not impact the bleeding rate in comparison to the continued therapy according to the studies of Miclotte *et al*. (10) and Lababidi *et al*. (13). However, the discontinuation of the new oral anticoagulants therapy leads to higher bleeding scores during the procedure (10) requiring additional hemostatic measures after the dental extraction (14). Another complication of the alteration of the DOACs therapy is the delayed bleeding mentioned in several studies, requiring additional hemostatic measures. Among the limitations of this review, only two studies (10,13) compare the complications of the DOACs discontinuation. And in both studies, only a small number of patients are treated (40 patients in total). According to the study of Nathwani and Wanis (20), local hemostatic measures allow to manage appropriately patients under DOACs after a dental extraction and the discontinuation of this anticoagulant treatment for the dental extraction should be decided by the prescribing physician. The present review has the same weakness as the study of Caliskan *et al*. (21). In fact, as in the studies included for the review (10,13), both reviews do not consider the short half-life of the DOACs and the time period between intake and tooth extraction. Despite the limitations of the number of studies including the discontinuation of DOACs for a simple dental extraction, further studies should include comparison of the discontinuation of DOACs and VKAs before undergoing dental extraction, in order to have more information about the postoperative complications of both oral anticoagulants for the dental clinicians. Furthermore, the lack of studies and evidence prevents us from being able to say whether the discontinuation of DOAC therapy before an invasive dental procedure (in the present review, simple dental extraction) will help reduce the postoperative complications. However, all the published studies concerning the discontinuation of the DOAC therapy show the same results and many studies are still in progress on this subject.

In conclusion, the most frequent postoperative complication for patients under DOACs and patients under VKAs after a simple dental extraction is minor bleeding: immediate or delayed. Patients under new oral anticoagulant treatment and patients not under anticoagulant have the same postoperative bleeding risk after a dental extraction. DOACs seem to be a safe drug and do not require the discontinuation/alteration of the therapy for a simple dental extraction. Further studies are required to determine if surgical extraction and other dental surgical procedures may require an alteration of the DOAC regimen.
